# Developing One Health Systems: A Central Role for the One Health Workforce

**DOI:** 10.3390/ijerph20064704

**Published:** 2023-03-07

**Authors:** Paulo Ferrinho, Inês Fronteira

**Affiliations:** Global Health and Tropical Medicine (GHTM), Institute of Hygiene and Tropical Medicine, NOVA University of Lisbon, 1349-008 Lisbon, Portugal

## 1. Conceptualizing One Health

The health of people, wild and domesticated animals, and natural living systems is syndemically connected, and this interplay is a pillar of the concept of One Health [1]. It has been acknowledged since immemorial time in the indigenous cultures of most continents [2,3].

The scientific roots of this concept can be traced back to Hippocrates and linked with Edward Jenner (1749–1823), Louis Pasteur (1822–1895), Rudolph Virchow (1821–1902), Robert Koch (1843–1919) and William Osler (1849–1919). Twentieth-century pioneers in this field include the USA’s former Assistant Surgeon General James Steele (1913–2013), who organized and developed the first veterinary public health program within the Center for Disease Control (CDC) in the 1940s and was responsible for the official inclusion of veterinarians in the US Public Health Service, beginning in 1947 [4]. The early efforts, since the 1950s, of Rachel Carson (1907–1964) to raise awareness of environmental issues have contributed to an appreciation of the health of the environment as an integral component in the One Health concept (although formal inclusion of the environmental context in One Health did not occur until 2008) [5]. William Osler is credited with coining the term “One Medicine” [6], but it was Calvin Schwabe (1927–2006), a public health veterinarian and parasitologist, who comprehensively reinvented the concept of “One Medicine” in 1976, thereby fully acknowledging the close systemic interaction of humans and animals for nutrition, livelihood and health [7,8]. For some time, the term “One Medicine, One Health” was utilized, before eventually becoming “One Health” during 2003–2004 [9,10].

Some recent initiatives, including the ‘One World, One Health symposium’ in 2004, which adopted the “Manhattan Principles”, and the 2019 Berlin conference on ‘One Planet, One Health, One Future’, which approved the “Berlin Principles on One Health”, have contributed to the revival and systematization of the concept, building on notions of ecosystemic health and integrity and addressing emergent issues, such as pathogen spillover, climate change, and antimicrobial resistance [1].

One Health is defined as the collaborative effort of multiple disciplines who work locally, nationally and globally to attain optimal health for people, animals and our environment [11,12].

Despite growing acceptance of One Health, it has been criticized for a disproportionate emphasis on public health, a narrow professional focus on physicians and veterinarians, excessive focus on emerging zoonotic diseases, inadequate incorporation of environmental concepts and expertise, and insufficient integration of social science and the behavioral aspects of health and governance which acts to sustain scientific barriers between human, animal and environmental sectors [1].

## 2. The Concept of One Health Systems

We argue that One Health systems have been emerging consistently and with subtlety, not following the definition of a traditional health system but rather the concept of a loose system for health, straddling sectors, countries and their institutions, organizations, resources and civil society whose purpose is to improve One Health; this may be through efforts to influence its determinants.

## 3. Is It Legitimate to Talk of One Health Systems?

If the adoption of a One Health nomenclature in the published literature is recent, dating only as far back as 2004 [13], the emergence of the term One Health systems is even more recent, dating from 2017 [14].

One Health systems are understood as “the wide range of roles and responsibilities and (…) interactions among diverse actors seen in systems that deal with (…) multi-sectoral challenges”. One Health systems build on the development of processes “that can be used to evaluate existing systems, foster synergies across agencies and improve multi-sectoral preparedness, detection, and response to complex One Health challenges” [15]. A recent publication, acknowledging that “health efforts are often siloed and frequently sectoral”, calls on governments to support “dedicated persons to promote the implementation of roadmaps” with the priority “to engage sectors in a systems-based approach at national/subnational levels” to strengthen One Health systems in countries [16].

Countries have now a range of One Health tools to assess “capacities within and between sectors, plan and prioritise activities, and strengthen multisectoral, One Health coordination, communication, and collaboration” [17]. These applications share a systemic focus [18]. In describing one of these tools, Errecaborde et al. (2017) refer to “the importance of a One Health systems approach to zoonotic disease problems” [14]. 

The same research group reports that their One Health Systems Mapping and Analysis Resource Toolkit (OH-SMARTTM) has been used to strengthen One Health systems in several countries. 

Multilateral organizations have since 2017 referred to human–animal–environmental health systems [19], One Health systems for zoonotic diseases [20], and One Health systems for early detection and surveillance of pathogen spillovers and anti-microbial resistance [21]; they have called for “Sustained One Health systems at the national, regional and global level through country investments in strengthening contributions to One Health and biodiversity, and its ecosystem services, environmental health, soil/land, water, food safety and the sustainability of agrifood systems” with the cross cutting objective of “strengthened One Health systems including enhanced One Health workforce capacities, investment and infrastructure at all levels” [22]. A World Bank Report addresses the expenditure required in all developing countries to build and operate One Health systems and the expected benefit of these systems to the global community [23]. Action track 1 of the ‘One Health Joint Plan of Action’ (2022–2026) calls for effective One Health coordination and integration of a system to develop One Health systems [24]. 

We wish to build on these recent conceptual developments to propose an understanding of One Health systems based on a building blocks approach.

## 4. Building Blocks for One Health Systems’ Development

Building blocks offer basic capabilities that can be used to design and construct solutions. They offer modular components that may be relied on to work together and grow coherently as the pieces making up a system. Using these blocks helps to focus design efforts on the important questions of what content to address, how to present it to policy makers, and how to manage it effectively. They facilitate the development of services across organizations and borders. Systems are built up from collections of building blocks, so most building blocks must interoperate with other building blocks. Although “building blocks alone do not constitute a system any more than a pile of bricks constitutes a functioning building”, “it is the multiple relationships and interactions among the blocks—how one affects and influences the others, and is in turn affected by them—that convert these blocks into a system”; building blocks’ frameworks are valuable because they create a common language and a shared understanding [25].

The building blocks concept has been adapted by the World Health Organization (WHO) to apply to health systems. The WHO conceptualized health systems based on the following six building blocks: (i) service delivery, (ii) health workforce, (iii) health information systems, (iv) access to essential medicines, (v) financing, and (vi) leadership/governance [26]. Since its launch in 2007, this framework has been widely considered as the basis for national and global action plans to improve health outcomes. Since the release of the framework, research has identified that “the WHO health system framework is instrumental in strengthening the overall health system, and as a catalyst for achieving global health targets such as the Sustainable Development Goals” [27]. Some authors argue that it can be improved by making four amendments: integrating the missing “demand” component; incorporating an overarching, holistic health systems viewpoint; explicitly including considerations of decision-making and power; and including scope for interactions between components [28]. Others question “whether the framework is sufficient in strengthening health systems so that they are resilient enough to respond to global health emergencies, as well as the ever increasing complexity of the ongoing challenges of health systems”, and advocate that “two additional domains be added to the WHO’s six conventional building blocks framework: (1) a meaningful inter-sectoral collaboration and (2) a functioning global health surveillance and response system” (https://blogs.bmj.com/bmj/2021/04/14/we-must-redesign-the-whos-building-blocks-to-create-more-resilient-health-systems-for-the-future/, accessed on 28 February 2023).

A World Bank Report has addressed “foundational building blocks to develop One Health interventions that may be implemented at varying levels of specificity (e.g., for a particular pathogen prioritized for preparedness) or broadness (e.g., any pathogens that could be present in or introduced to a country)”. These building blocks, may be presented separately in distinct stages (prevention, detection, response and recovery); however, effective interventions rely on the individual pieces coming together to support dynamic public health systems in practice, with strong connections within and between the systems providing continuous feedback loops for optimal functioning (for example, findings obtained during outbreak investigations in the response phase may directly inform risk assessment and management to guide prevention efforts). These building blocks include (i) stakeholders, roles, and responsibility; (ii) financial and personnel resources; (iii) communication and information; (iv) technical infrastructure; and (v) governance [23]. Their overlap with some of the building blocks proposed by WHO is striking, and the workforce is an element shared by both frameworks.

The workforce building block is central to meeting One Health goals that depend largely on the knowledge, skills, engagement and deployment of the people responsible for organizing and delivering services. Many countries, however, lack the human resources needed to meet basic human, animal or environmental healthcare needs, and may be distressed by the thought of diverting scarce human resources to address the needs of other sectors. Integrated One Health interventions must be sensitive to these concerns. The formulation of One Health national policies and plans in pursuit of human resources for One Health development objectives thus requires sound information and evidence. Against this backdrop of increasing demand for information, knowledge-building and databases on the human, animal and environmental health workforce require coordination across sectors. Various permutations and combinations of what constitutes the One Health workforce (e.g., public–private; full–part time; paid–voluntary; service or education; front-line, intermediate or advanced level) may exist according to a country’s unique situation, and its means of financing and monitoring.

However, collaboration across the interface of humans, animals and the environment is a core element of global workforce transformation initiatives and efforts. The World Bank report previously referred to is littered with examples of such collaborations [23]. 

The FAO, UNEP, WHO, and WOAH’s ‘One Health Joint Plan of Action (2022–2026)’ acknowledges “professional segregation with limited cross-sectoral working, inadequate representation of some sectors, disjointed legislative schemes, a lack of data sharing and transparency, an absence of multisectoral coordination mechanisms, siloed budgets and decision-making processes, and a lack of robust regulatory frameworks, legal support, mandates and enabling policies are additional barriers hindering the effective implementation of One Health (…) One Health requires (…) effective governance rooted in transdisciplinary and multisectoral principles and appropriate legislation, stakeholder and community engagement, and the integration of the concept into education in related disciplines”. Workforce and professional issues are identified at four of the six Action Tracks of the Plan (Table 1). 

One Health principles apply equally “well to protecting nations’ public health” and “to clinical medical and surgical research (comparative medicine)” and practices, e.g., “in the fields of cancer, cardiovascular disease, orthopedic conditions, obesity, and many others”. Far more One Health interest is apparent in public health academic communities than within clinical health academic groups, and it is almost unheard of within practicing veterinary and human medical communities. One Health needs to move beyond public health to include clinical medical/surgical research and practice, and to mobilize professional groups other than physicians and veterinarians in order to avoid travelling “the path of ‘some health’ and not One Health” [29]. To achieve this, One Health must be embraced by a wider scope of researchers and practitioners, including “medical doctors, veterinarians, nurses, agronomists, nutritionists, psychologists, historians, anthropologists, statistics, biologists, dentists, conservationists, engineers, artists, and dancers” [2,12].

Driven by climate change, emerging and reemerging zoonosis, pandemics and global syndemics, One Health is becoming a fundamental theme in Global Health debates. One Health demands multi-professional attention and efforts to advance research, training, and practice. Many of the critical issues in this area are cross-disciplinary in nature and have been discussed conceptually; however, they remain short of a unified conceptual viewpoint and neglect practical implementation bottlenecks such as workforce issues. Workforce issues tend to focus on medical and veterinary staff, neglecting other workforce categories. The capacity to successfully address threats to human, animal, and environmental health requires an adequate workforce. Yet, capacity building and workforce development within a One Health framework remain challenging for many reasons, including conflicting priorities, sector-specific policies and funding, and an absence of trust and interactions across sectors. Accordingly, previous attempts to build the workforce capacity of the One Health have traditionally lacked the attributes necessary to break existing silo mentalities and ensure the continued and systematic coordination of the One Health workforce development process. 

This Special Issue, 10 years after the wide acceptance of the term One Health, calls for papers that go beyond narrow conceptual approaches and professional understandings, and requests that contributors examine important workforce issues through the broad lens of One Health, as presented in Table 1. To provide a solid evidence base and background to the theme, the series will feature commissioned as well as independently submitted articles that will contribute to innovative thinking on One Health systems’ workforce, thereby contributing to the achievement of the Sustainable Development Goals and to universal health coverage. The deadline for submissions is 15 October 2023. Manuscripts should respect the Journal Guidelines for contributors (available at guide for authors and other relevant information for submission of manuscripts is available on the Instructions for Authors page) and mention this call for papers in the cover letter. All submissions will be reviewed by peers.

## Figures and Tables

**Table 1:** Workforce-related Action Tracks of the FAO, UNEP, WHO, and WOAH’s ‘One Health Joint Plan of Action (2022–2026)’ [24].

Action Track	Scope	Activities and Deliverables
AT1.2	Generate mechanisms, tools, and capacities to establish a One Health competent workforce and to facilitate One Health work	1.1.2 Define One Health institutional and workforce capacities and develop methodologies and tools to assess national One Health performances and identify needs: Defining One Health competencies and capacities at institutional and individual levels Mapping and integrating existing methodologies and tools, and new methodologies, tools and pilot tests for: ◦ national capacities for One Health and the performance of systems at the human–animal–plant–environment interface ◦ One Health competencies ◦ Workforce learning needs assessment ◦ Supporting the application of the tools and assessments provided ◦ Identified learning needs ◦ Identified opportunities to strengthen One Health’s coordination
		1.2.2 Facilitate One Health capacity building, including workforce development in all relevant sectors: Mapping of existing opportunities, resources and curricula at global, regional and national levels Definition of access and selection criteria and processes evaluation of capacity-building programs Mechanisms to build synergies and avoid duplication in capacity-building delivery Competency-based frameworks, training programs, courses, plans and e-learning resources Workforce development tools Job descriptions for One Health professionals Simulation exercises to build and strengthen One Health competencies Monitoring and evaluation tools to assess capacity building
		1.2.3 Support and promote the next generation of One Health practitioners, researchers and technical officers Internships, placements, mentorship schemes and a competency framework for junior One Health practitioners, researchers and technical officers
AT3.1	Enable countries to develop and implement community-centric and risk-based solutions to endemic zoonotic, neglected tropical and vector-borne disease control using a One Health approach involving all relevant stakeholders	3.1.1 Provide integrated guidance and resources to countries to help build capacity and resilience, empower communities and increase engagement and awareness of endemic zoonotic, neglected tropical and vectorborne disease prevention, diagnosis, control and treatment: On request, integrated multisectoral training of professionals, paraprofessionals and laboratory staff working on the health of humans, animals and/or the environment
		6.2.10 Promote the adoption of climate-smart and environmentally sound health systems: Health workforce interventions
AT6.4	Create an interoperable One Health in-service training program for environment, medical and veterinary sector professionals	6.4.1 Develop advocacy training and tools for environmental decision makers and professionals to influence decision makers in other sectors: Advocacy training and tools developed and used by relevant sectors
		6.4.2 Develop and roll out a national environment sector needs assessment tool to benchmark institutional and individual capacity to participate interoperably in all aspects of One Health, in support of the Field Training Programme for Wildlife, Environment, Biodiversity and Ecosystems Professionals (FTPWEBE)
		6.4.3 Develop an interoperable One Health training course (FTP-WEBE) for in-service professionals as a complement to the Field Epidemiology Training Programme (FETP), Field Epidemiology Training Programme for Veterinarians (FETPV) and Field Epidemiology and Laboratory Training Programme (FELTP), targeting professionals in ministries responsible for natural resource management (wildlife, biodiversity, ecosystems, environment), climate and other environmental issues: Interoperable environmental sector One Health training modules and course developed Interoperable environmental sector One Health training delivered on biodiversity, ecosystems and wildlife Environment sector professionals understand how to contribute to One Health at national and subnational level Environment sector has the capacity to influence One Health policy and identify and implement environmental sector priorities as part of national and subnational One Health programs National One Health policies and priorities reflect the mandates and interests of environment ministries and are expanded beyond zoonoses, anti-microbial resistance and food safety
		6.4.4 Develop and ensure the inclusion of training for in-service medical, public health and veterinary professionals on the importance of and interlinkages between biodiversity conservation, links between health and the environment, how environmental destruction contributes to disease emergence, and the importance of integrating the environment sector into One Health collaborations: At least three training modules developed to include the environment (biodiversity and ecosystem health) and its importance and interlinkages One Health collaboration across sectors and interfaces Emphasis on the impact of diseases on wildlife populations and conservation

## Data Availability

Not applicable.
